# Postoperative pathology confirmed perianal plexiform neurofibroma: a case report

**DOI:** 10.3389/fmed.2026.1842411

**Published:** 2026-07-30

**Authors:** Zhong Bo Wu, Juan Li, Min Wu, Ru Song Tian, Song Qiao, Yong Tian

**Affiliations:** 1 Brain Disease Research Center, Tongren Municipal People’s Hospital, Tongren, Guizhou, China; 2 Departments of Pathology, Tongren Municipal People’s Hospital, Tongren, Guizhou, China; 3 Departments of Radiology, Tongren Municipal People’s Hospital, Tongren, Guizhou, China; 4 Department of Gastrointestinal Surgery, Tongren Municipal People’s Hospital, Tongren, Guizhou, China

**Keywords:** case report, neurofibromatosis type 1, pathology, perianal, plexiform neurofibroma

## Abstract

**Background:**

Plexiform neurofibroma (PN) is extremely rare in the perianal region, primarily attributed to the lack of thick nerve trunks in the perianal area, and is closely related to neurofibromatosis type 1 (*NF1*).

**Case summary:**

Here we described a 21-year-old man who was admitted to the hospital with a chief complaint of a painless mass on the left perianal area for 10 years. Physical examination revealed a nodular cystic-solid mass approximately 6.0 cm × 3.0 cm in size in the left perianal area, with an irregular shape, soft texture, good mobility, and no obvious tenderness; no abnormalities were found during rectal examination. Preoperative pelvic magnetic resonance imaging revealed a 55 × 27 mm soft tissue lesion in the left ischial region, with radiological characteristics typical of nerve-derived tumors. The patient underwent complete surgical resection of the tumor, and postoperative histopathological analysis confirmed the diagnosis of PN without malignancy.

**Conclusion:**

Complete surgical resection was the cornerstone, and histopathological confirmation was crucial. Long-term surveillance should be regarded as a means to prevent tumor recurrence or metastasis.

## Introduction

PN was typically present as characteristic lesions of the genetic disorder NF1; only 30 ~ 60% of *NF1* patients were accompanied by PN (1), and the lesions are predominantly found in the head and neck, trunk, and extremities (2–3). However, it is extraordinarily rare to have sporadic, isolated perianal PN without the systemic NF1 phenotype.

*NF1* is a common autosomal dominant genetic disorder and is located on chromosome segment 17*q*11.2, encoding a protein with tumor suppressor function, characterized by benign tumors (neurofibromas) (4). Individuals with *NF1* present with several hallmark clinical features, such as café-au-lait macules, freckling in the axillary and inguinal regions, skeletal abnormalities, and ocular Lisch nodules (5).

The distinct pathological variant of *NF1* arises from Schwann cells (SCs) within the perineural sheath and is classified as a benign tumor of neural origin (6). Loss of *NF1* heterozygosity in SCs leads to the formation of PN benign yet complex neoplasms involving multiple nerve fascicles and comprised of a myriad of infiltrating stromal and immune cells (7).

Although these tumors can develop across various anatomical locations, neurofibromas are categorized into three primary forms based on clinical and histopathological criteria: localized, diffuse, and plexiform. The plexiform subtype is notable because of its multinodular structure, web-like or branching growth along nerve pathways, and predisposition to malignant transformation (8).

PN is an irregular, thick, and non-circumscribed tumor of the peripheral nerve sheath that can involve multiple nerve fascicles. This disease is a rare entity and occurs in approximately 5 ~ 15% patients with *NF-1*. These are slow-growing, painless, and locally infiltrating tumors. The diffuse and soft nature of plexiform neurofibroma is often compared to “a bag of worms” and is difficult to distinguish from a vascular malformation or a lymphangioma (9). PN demonstrates diffuse cylindrical enlargement of multiple fascicles of a nerve, including the nerve branches leading to a diffuse mass of thickened nerves.

Due to the rarity of PN localization, comprehensive epidemiological studies are currently lacking, and the exact incidence rate remains uncertain. Considering the intricate anatomy of the perianal region, which is closely associated with critical structures, such as the sciatic nerve and anal sphincter, complete surgical excision poses substantial technical challenges and carries a high risk of recurrence. Thus, we report this case, which has considerable clinical value and warrants thorough documentation and dissemination.

This case report aimed to present a rare case of a perianal PN; the patient was a 21-year-old man who suffered from a painless mass in the left perianal area and was treated in Tongren Municipal People’s Hospital in January 2025. Here, the medical history, clinical symptoms, signs, laboratory results, imaging data, and histopathological examination results were reported as follows. The patient was informed that the data from his case would be submitted for publication, and he agreed.

## Case presentation

A 21-year-old man presented to our hospital with a painless mass in the left perianal region that had grown over a decade. The patient had no café-au-lait spots, axillary or inguinal freckling, Lisch nodules, skeletal abnormalities, and no history of any diseases, family history of hereditary diseases, digestive diseases, infectious diseases, smoking, alcohol consumption, drug abuse, and so on.

A clinical examination revealed that his height is 178 cm, weight is 76 kg, and his body mass index (BMI) is 24.61 kg/m^2^. Vital signs, laboratory tests, and electrocardiogram (ECG) were all normal.

Physical examination revealed that at a distance of 1.0 cm from the anal sphincter, a 6.0 cm × 3.0 cm × 3.0 cm nodular cystic-solid mass on the left perianal area, with an irregular shape, soft texture, good mobility, and no obvious tenderness. Rectal examination revealed no abnormalities. Skin and related regional lymph node examinations indicated that no enlarged lymph nodes were palpable in either inguinal region, and there were no freckles or coffee-ground macules in the axillary area.

**Figure 1:**
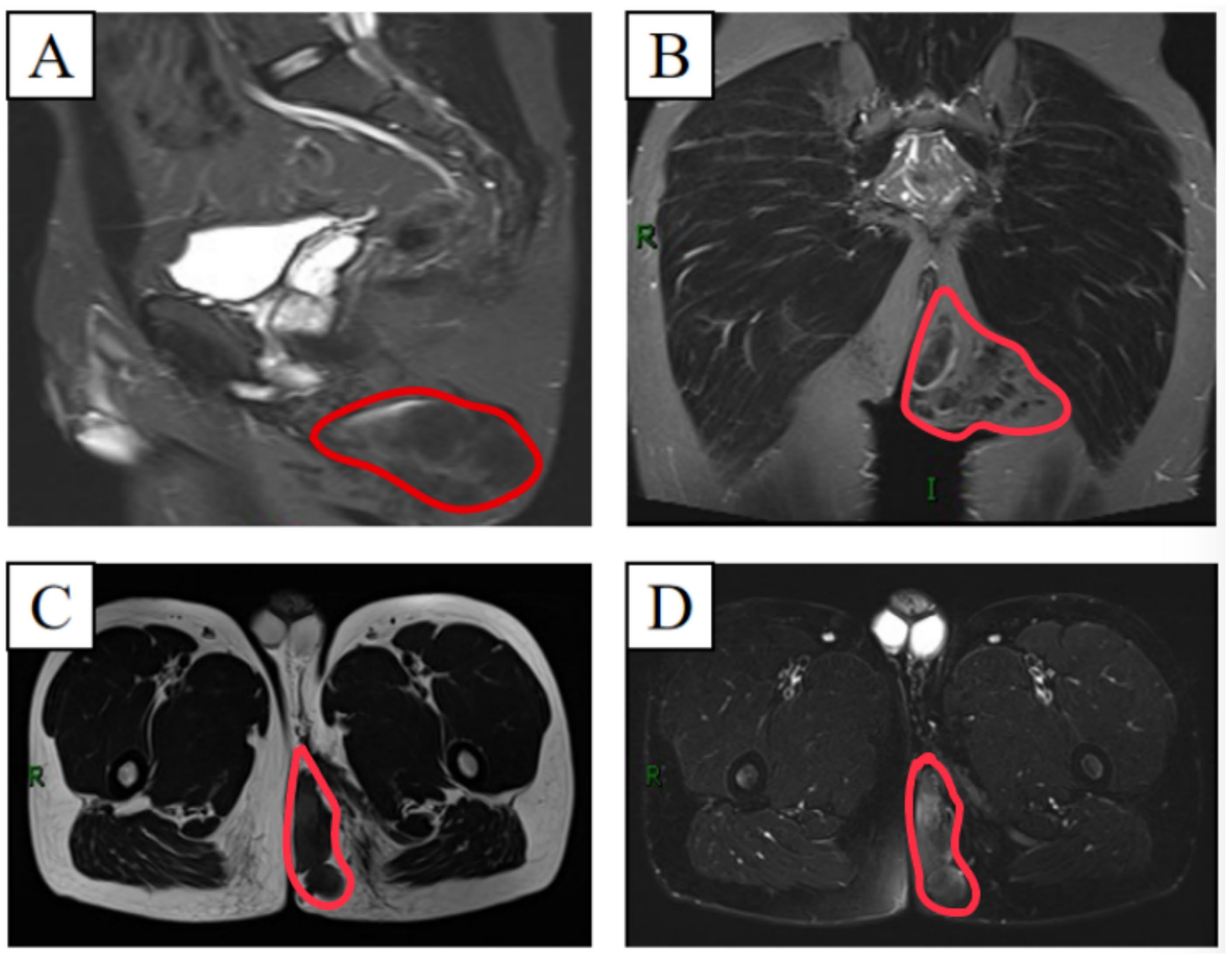
**(A–D)** Pelvic MRI indicated that the perianal tumor is located in the left gluteal cleft (red circle). Long T1 and slightly prolonged T2 signals, and the fat-suppressed sequence (T2WI-FS) showed a mildly high signal. Patchy long T2 signal shadows surround the lesion, and diffusion-weighted imaging showed a mildly high signal.

Pelvic magnetic resonance imaging (MRI) revealed a circular space-occupying lesion near the left ischial tuberosity, with long T1 and slightly prolonged T2 signals, and the fat-suppressed sequence (such as T2WI-FS) indicated a mild high signal; the maximum diameter was approximately 55 mm × 27 mm. Patchy long T2 signal shadows surround the lesion, and diffusion-weighted imaging shows a mild high signal. The corresponding apparent diffusion coefficient map showed a mild high signal, suggesting a lower degree of water molecule diffusion limitation, which did not match the manifestation of high-cell density malignant tumors. Based on the imaging characteristics, the lesion signal characteristics were consistent with those of a neurogenic tumor. MRI findings demonstrated that the lesion was located in the left anal canal soft tissue, extending along neural pathways with poorly defined margins and exhibiting heterogeneous enhancement on contrast-enhanced scans, features highly suggestive of a neurogenic origin (Figures 1A–D).

We organized a multidisciplinary team (MDT) discussion preoperatively, preliminarily diagnosed him with a perianal neurogenic tumor based on the physical examination and imaging report, and drew up an accurate surgical plan. The patient’s family should be fully informed of the relevant surgical risks, and they should sign the surgical consent form preoperatively.

The patient was positioned in the lithotomy position during surgery, which strictly adhered to the principles of sterility and tumor-free. A 3-centimeter spindle-shaped incision was performed in the left perianal region, designed to achieve complete resection of the mass and minimize recurrence risk. Upon sequential dissection through the skin and subcutaneous layers, a cord-like neoplasm was identified infiltrating along branches of the sciatic nerve, extensively adhering to surrounding adipose and fascial tissues, with indistinct anatomical demarcation. The tumor encased portions of the nerve trunk, resulting in distortion, thickening, and morphological deformation of the involved nerve fascicles; no significant cystic degeneration or necrosis was noted. The tumor lacked a well-formed capsule, exhibited an irregular contour, had a firm consistency, and demonstrated abundant vascularity. Careful dissection was required to preserve nearby critical neurovascular structures. The whole surgery lasted for 1.5 h. Blood loss was approximately 30 mL, without blood transfusion and intraoperative complications.

**Figure 2:**
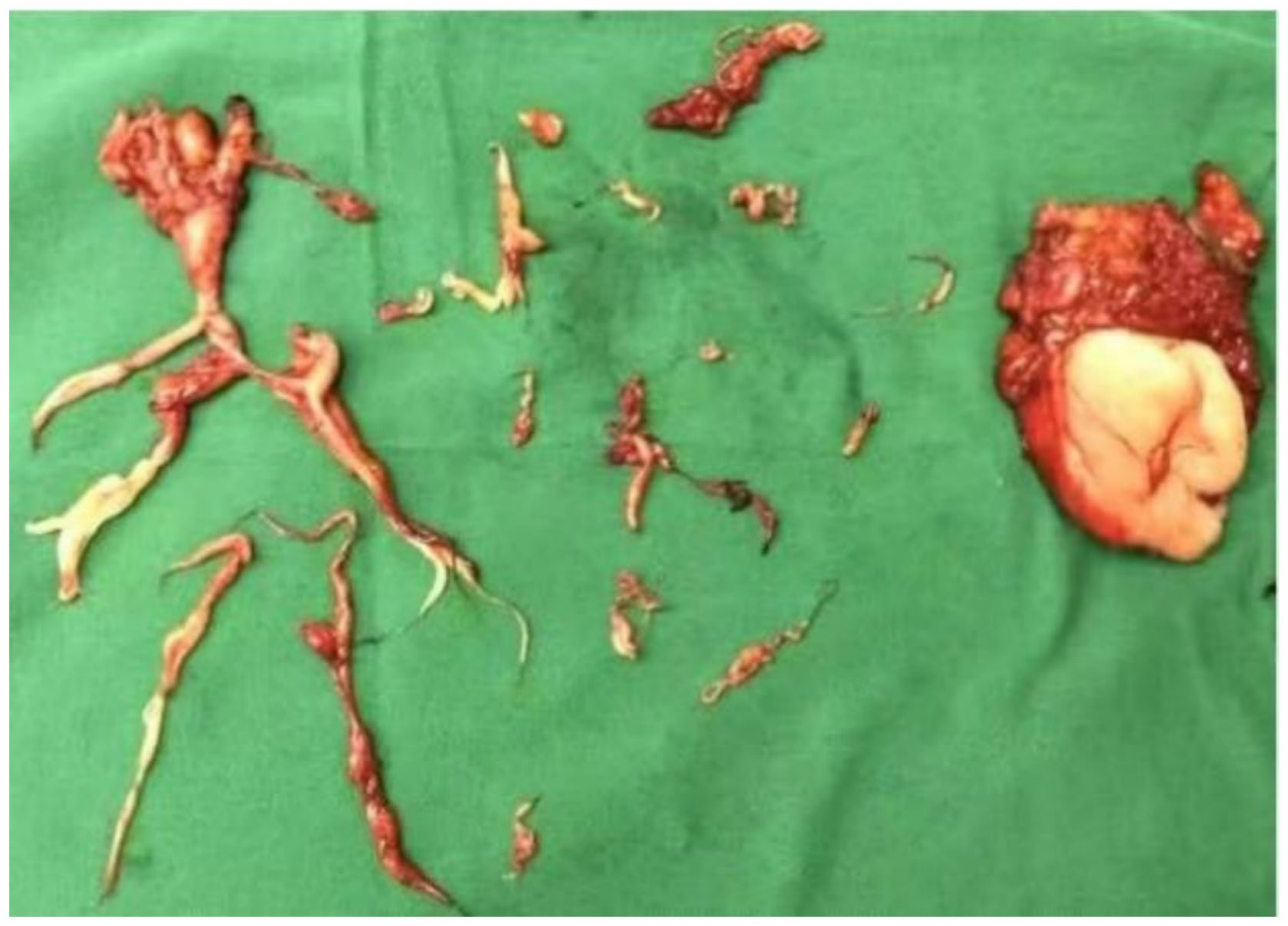
Resected specimen, macroscopic findings. The tumor specimen presented as a cord-like structure; the cut surface of the tumor was grayish-white, with an irregular shape, uneven surface, a medium-to-firm texture, and its appearance resembled “a bag of worms.”

On macroscopic examination of the post-resection specimen, a 7.0 cm × 5.0 cm × 2.0 cm tumor was identified, with an irregular shape, uneven surface, and a grayish-white cut surface, with a medium-to-firm texture, and interspersed with focal translucent and gelatinous areas. The gross appearance resembled “a bag of worms” configuration, indicating a plexiform architecture arising from the surrounding peripheral nerve network (Figure 2).

**Figure 3:**
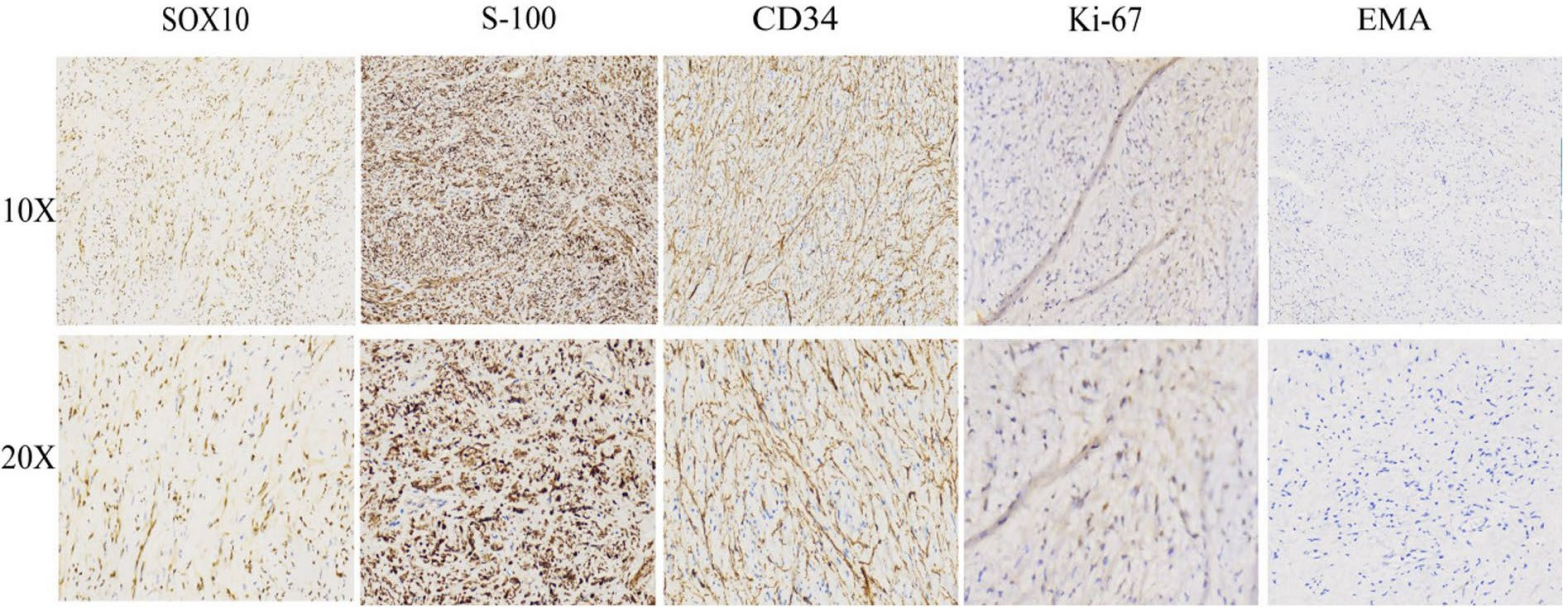
Immunohistochemical examination: S-100 and SOX10 are diffusely and strongly positive; CD34 stromal cells are positive; NF shows intratumoral axonal staining; Ki-67 proliferation index is extremely low (2%); EMA tumor cells are negative.

We conducted a histopathological analysis of the postoperative resected specimens. Histologically, low-power magnification microscopy demonstrated multiple nodular “plexiform like” structures can be seen, with a large number of neurofibroma nodules wrapped by the nerve bundle membranes; these nodules varied in size, appeared as round or twisted cord-like structures, scattered in loose connective tissues. High-power microscopy revealed spindle-shaped Schwann cells within the tumor cells. The cell nuclei were elongated and wavy in shape, with scarce cytoplasm. The stroma appeared as a loose, pale blue, mucus-like matrix interspersed with collagen fibers. No obvious signs of nuclear fission or necrosis. Immunohistochemical analysis showed that SOX-10 and S-100 proteins were diffusely and strongly positive, further confirming the Schwann cell lineage. Focal expression of CD34 in certain regions, suggesting potential mesenchymal interactions. The leucocyte antigens were positive [SOX10, S-100, CD34, Ki-67 (2%)] and negative (SMA) on the tumor cells observed (Figure 3).

**Figure 4:**
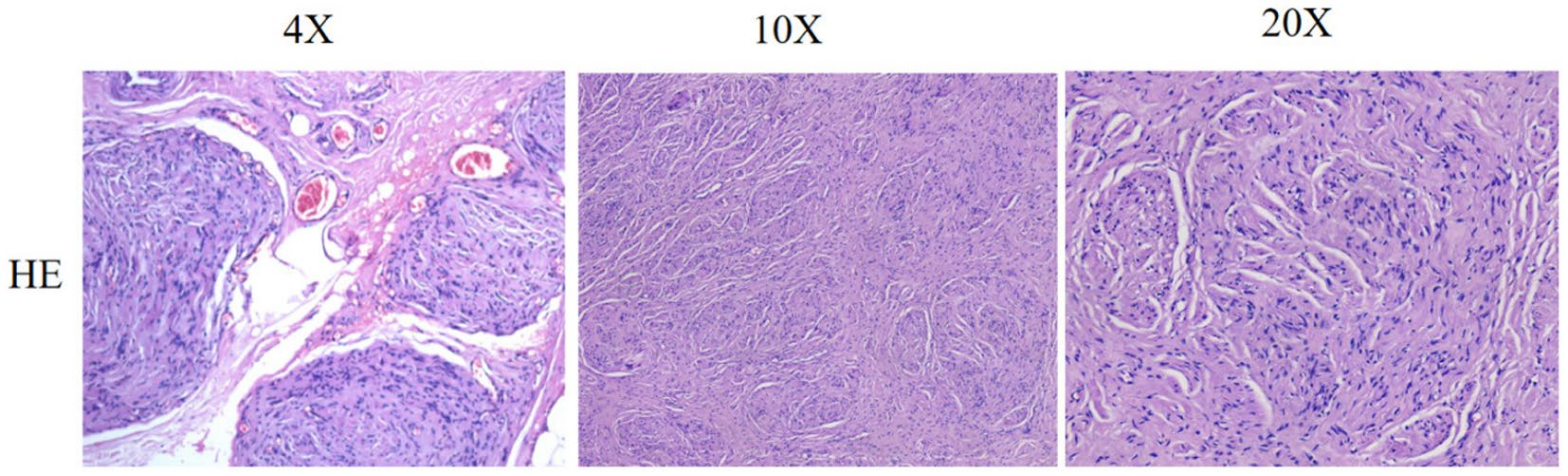
HE staining reveals that the tumor resembles a “worms” or “ropes” structure.

Hematoxylin and eosin (HE) staining revealed multiple neurofibromatous nodules; the tumor was composed of multiple dilated and twisted bundles of nerves, resembling a cluster of “worms” or “ropes” (Figure 4). Postoperative histopathological analysis and HE staining consistently confirmed the diagnosis of perianal PN.

The patient exhibited well-healing postoperative wounds, with anal sphincter function remaining intact and no signs of fecal incontinence. There was no anal stenosis or incision dehiscence. Regular follow-up evaluations are recommended at 6 ~ 12-month intervals to monitor the long-term outcomes.

## Discussion

Although PN generally occurs in the head, neck, trunk, and extremities, and extraordinarily rare in the perianal region. This case report describes a patient who presented with a painless mass in the left perianal area, without other clinical symptoms, such as café-au-lait macules, freckles in the axillary and inguinal regions, skeletal abnormalities, and ocular Lisch nodules. However, the postoperative pathological diagnosis was perianal PN.

Due to the special location of the lesion and the extremely low incidence rate, clinicians lack experience in diagnosing and treating this disease, and the preoperative misdiagnosis rate is high. Moreover, the tumor grows invasively, has abundant blood supply, and is adjacent to key anal canal anatomical structures related to defecation, posing great difficulties for surgical intervention. Therefore, surgery must be carefully balanced to achieve complete tumor removal while minimizing damage to sphincter integrity. Any inadvertent intraoperative trauma can result in irreversible fecal incontinence, profoundly impairing the daily lives of patients. The surgical approach aims to achieve total tumor removal while preserving neurological integrity and maintaining bowel continence.

It is worth noting that the patient exhibited atypical clinical symptoms of *NF1* and presented solely with imaging manifestations, making early diagnosis rather complicated. Considering the limited size and superficial location of the tumor, surgical excision was considered appropriate. The occurrence of PN in this anatomical location is rare, and resection is challenging owing to the intricate surrounding anatomy, including proximity to critical neural and vascular structures, such as the sciatic nerve and anal sphincter complex. Preoperatively, carefully analyze imaging examinations, accurately determine tumor infiltration boundary tubes, and establish objective standards for the resection range. Intraoperative refinement uses normal tissue layers as anatomical landmarks; the tumor tissue is dissected segment by segment, with complete removal of all proliferating and infiltrating tumor-like tissues to confirm no tumor residue.

Preoperative evaluation of surgery and medical management should be based on expert opinions from a multidisciplinary team. In general, surgery is the optimal choice if the PN can be resected without significant morbidity. Due to the connection between PN and nerves, this is generally a challenging task in most cases. The indications and scope of surgery need to be adjusted according to the extent, location, growth rate, radiological characteristics, and overall health status of the tumor. The indications for surgical intervention include actual or imminent nerve damage or impact on important structures. Relevant indications for surgical intervention may include pain, disfigurement, and the aim of improving activities of daily living. For progressive tumors that may endanger critical structures or cause functional decline or disfigurement, debulking surgery may be considered.

When the perianal PN does not have adjacent invasion or distant metastasis, surgical resection is the standard treatment for managing PN (10–11). However, when the tumor exhibits invasive growth and infiltrates adjacent blood vessels, critical nerves, or surrounding organs, complete resection is unfeasible owing to substantial technical and safety challenges. Aggressive wide excision in such cases may lead to extensive tissue defects, significant functional deficits, and an elevated risk of postoperative complications. Consequently, debulking surgery, a more conservative strategy, is commonly adopted to reduce tumor volume, alleviate symptom burden, and improve patient quality of life (12–14). Although reduced surgery can control disease progression to a certain extent, the recurrence rate is significantly higher because of the presence of residual lesions postoperatively. Research has shown that the postoperative recurrence rate of patients with incomplete resection can reach 45%, whereas that of those who undergo radical resection is approximately 20% (15). Various factors affect postoperative recurrence, including whether the tumor is located in deep or critical anatomical areas, the rapid progression of the disease in young patients owing to its insidious development, and specific resection strategies and techniques adopted intraoperatively.

The postoperative histopathological and immunohistochemical findings provided definitive molecular evidence to confirm the final diagnosis of perianal PN in this case. HE staining revealed that the lesion presents as a proliferation of plexiform nerve bundles, exhibiting characteristic “a bag of worms” changes of PN. Microscopically, the tumor is composed of proliferating spindle-shaped Schwann cells and fibrous stroma, arranged in a cord-like or cluster-like invasive manner. Without obvious signs of nuclear fission or necrosis, and histological changes related to malignant peripheral nerve sheath tumors were absent. The immunohistochemical marker results indicated that the tumor cells were diffusely and strongly positive for SOX10 and S-100, both of which were characteristic markers of neural crest-derived Schwann cells and could specifically confirm that the lesion originated from the peripheral nerve. CD34 was positively expressed, consistent with the expression characteristics of the interstitial blood vessels and fibrous tissues in plexiform neurofibromas. EMA expression was negative, which effectively excluded epithelioid-origin tumors and other perianal soft tissue lesions. The Ki-67 proliferation index of the tumor was merely 2%, and the risk of local progression and malignancy was extremely low; it was consistent with the biological characteristics of benign tumors. Based on the HE staining characteristics and immunohistochemical indicators, we have confirmed that the case is a benign perianal PN.

The accurate diagnosis of postoperative PN was made based on the pathological examination report, requiring experienced pathologists and the application of immunohistochemistry and cytogenetics. In addition, pathologists need to differentiate these diseases from other conditions, such as perianal solitary fibroma, lipoma, schwannoma, hamartoma, lymphoma, lipoblastoma, Legius syndrome, McCune–Albright syndrome, Noonan Syndrome, and neurofibromatosis type II (NF II). If a difficult diagnosis is encountered, senior pathologists should be consulted, and genetic testing can be performed if necessary.

Although the patient underwent surgical intervention and achieved the therapeutic goal to a certain extent, considering the characteristics of PN, such as multiple lesions, blurred boundaries, indistinct anatomical demarcation, and interwoven growth with normal tissues, a complete cure is difficult to achieve solely through surgery. Targeted therapy provides a breakthrough for the long-term management of PN, especially with the rapid development of individualized precision medicine (16).

Individuals with *NF1* can be significantly impacted by NF1-related complications, and targeted therapies are emerging. Currently, the inhibition of MAPK kinase (MEK) inhibitors selumetinib and mirdametinib are targeted therapy drugs suitable for NF1-PN patients aged 2–17 years who have symptoms and are not eligible for surgery (17–20). Clinical trials have shown that selumetinib can significantly shrink tumors in most patients, with a high objective response rate, pain relief, improvement in motor function, and overall improvement in the quality of life (21), and can be used for preoperative neoadjuvant treatment to reduce tumor volume and improve the operability of subsequent surgery (17).

There are numerous surgical challenges in this perianal PN case. First, this disease is rare and prone to misdiagnosis, making it difficult to accurately assess the depth and extent of tumor invasion, which increases the difficulty of surgical planning. Second, the tumor shows diffuse and invasive growth, without a complete capsule, and has unclear boundaries with small nerves and connective tissues around the perianal area, making it difficult to accurately define the resection range and increasing the risk of residual lesions or excessive resection. Third, the perianal area is adjacent to the core functional structure of the internal and external anal sphincters, and radical resection can easily damage the sphincter and cause defecation dysfunction, while conservative resection also carries the risk of recurrence, and the contradiction between functional protection and lesion resection is particularly prominent. Fourth, the surgical area is narrow, and the tissues are densely adhered around the perianal area, making a delicate separation and operation extremely difficult.

Based on this case, we have summarized the experience and lessons learned in diagnosis and treatment. First, we conducted a comprehensive preoperative multidisciplinary assessment, combining imaging, biopsy, and anal function examinations, accurately identified the lesion, assessed the extent of invasion, and formulated an individualized function-preserving surgical plan. Second, followed the principle of prioritizing function and ensured sterility and tumor-free status; the visible lesions should be removed as thoroughly as possible while protecting the sphincter muscles. Third, fine intraoperative separation and meticulous dissection were necessary, distinguished between tumors, perianal nerves, and anal sphincters, avoided extensive electrocoagulation and excessive resection, protected the function of defecation, and reduced complications. Fourth, established a standardized long-term follow-up system, regularly reviewed imaging and anal control of defecation function, and promptly detected recurrences and intervened.

We conducted close follow-up via phone every 6 months; the patient has no discomfort symptoms such as perianal pain, recurrent lumps, anal stenosis, or fecal incontinence. His mental state, diet, sleep, and fecal movements were all normal. The tumor markers were also within the normal range. The pelvic CT scan showed no recurrence or metastasis of the tumor, exhibiting a stable clinical status.

## Conclusion

Perianal PN represents an uncommon subtype of neurogenic tumors that are frequently associated with NF1. Surgical management requires careful preservation of anal sphincter integrity to reduce the likelihood of postoperative functional deficits. It improved the differential diagnosis and functional preservation experience of perianal PN, providing valuable evidence for individualized and standardized clinical treatment of this disease. Continuous and systematic follow-up is crucial.

## Data Availability

The original contributions presented in the study are included in the article/supplementary material, further inquiries can be directed to the corresponding author/s.
