# Correction: Postoperative pathology confirmed perianal plexiform neurofibroma: a case report

**DOI:** 10.3389/fmed.2026.1953786

**Published:** 2026-07-31

**Authors:** Frontiers Production Office

**Affiliations:** Frontiers Media SA, Lausanne, Switzerland

**Keywords:** case report, neurofibromatosis type 1, pathology, perianal, plexiform neurofibroma

The wrong version of the manuscript was published. The article has been updated with the correct accepted version.

The original version of this article has been updated.

